# Effect of *Helicobacter pylori*-related chronic gastritis on gastrointestinal microorganisms and brain neurotransmitters in mice

**DOI:** 10.3389/fphar.2024.1472437

**Published:** 2024-12-06

**Authors:** Hai-Hua Liu, Tang-Tang Lin, Qi-Jia Zhang, Ling Zhang, Jin-Ying Fang, Ling Hu

**Affiliations:** ^1^ First Affiliated Hospital of Gannan Medical University, Gannan Medical University, Ganzhou, China; ^2^ Institute of Gastroenterology, Science and Technology Innovation Center, Guangzhou University of Chinese Medicine, Guangzhou, China; ^3^ The Second Affiliated Hospital of Zunyi Medical University, Zunyi Medical University, Zunyi, China

**Keywords:** Hp-related chronic gastritis, microorganism, neurotransmitter, metagenomics, metabolomics

## Abstract

The effects of *Helicobacter pylori* (Hp)-related chronic gastritis on gastrointestinal microorganisms or brain neurotransmitters are not fully understood. Here, this study selected SPF C57BL/6 mice to set up a Hp-related chronic gastritis experiment group and a blank control group, and used omics to explore the specific effects of Hp-related chronic gastritis on gastrointestinal microorganisms and brain neurotransmitters in mice. The Tyramine (TyrA) content in the female experiment group’s brain was considerably reduced compared to the female control group (*p* < 0.01), and TyrA was strongly correlated with 13 gastrointestinal microorganisms with significant differences, such as *Acinetobacter_baumannii* (*p* < 0.05). The His content in the male experiment group’s brain was significantly higher than that in the male control group (*p* < 0.05), and His was strongly correlated with four gastrointestinal microorganisms with significant differences, such as *Acinetobacter_baumannii* (*p* < 0.05). The Levodopa (DOPA) content in the female control group’s brain was significantly lower than that in the male control group (*p* < 0.05), and DOPA was strongly correlated with 19 gastrointestinal microorganisms with significant differences, such as *Achromobacter_xylosoxidans* (*p* < 0.05). The contents of L-Glutamine (Gln), L-Glutamine (GABA), Noradrenaline hydrochloride (NE), and Adrenaline hydrochloride (E) in the female experiment group’s brain were significantly lower than those in the male experiment group (*p* < 0.05), and Gln, GABA, NE, and E were strongly correlated with 41, 28, 40, and 33 gastrointestinal microorganisms with significant differences (*p* < 0.05), respectively. These results indicate that Hp-related chronic gastritis could affect gastrointestinal microorganisms and brain neurotransmitters in mice with certain gender differences, and the changes in brain neurotransmitters might be related to the changes in gastrointestinal microorganisms.

## Introduction

Gastritis has many pathogenic causes, and *Helicobacter pylori* (Hp), an infectious bacterium, is considered to be one of the main pathogenic factors of gastritis (Hanafiah and Lopes, 2020; Kalach et al., 2022). At present, Hp-related chronic gastritis has become highly prevalent (Rahman et al., 2020; Yin et al., 2022). Studies have revealed that Hp could destroy and change gastrointestinal microorganisms (Schulz et al., 2018; Zhao et al., 2019), and abnormal gastrointestinal microorganisms could cause diseases in the gastrointestinal tract and the other parts (Lopetuso et al., 2014; He et al., 2022). However, the specific effects of Hp-related chronic gastritis on gastrointestinal microorganisms are still unclear due to the lack of specific studies. Hp infection could be related to neurological diseases such as Alzheimer’s disease, Parkinson’s disease, and sleep disorders (Doulberis et al., 2021; Shen et al., 2017; Sung et al., 2022). Besides, Hp could be accompanied by various mental symptoms, such as anxiety and depression (Al Quraan et al., 2019; Suzuki et al., 2019). However, the specific mechanism has not been clarified. The pathogenesis of nervous system diseases and mental symptoms is complex, and some studies have shown that it is mostly related to neurotransmitters (Lovino et al., 2020; Nimgampalle et al., 2023). The effects of Hp-related chronic gastritis on neurotransmitters are still unclear due to the lack of research. The microbiota-gut-brain axis has been a hot topic in recent years and the effects of Hp-related chronic gastritis on gastrointestinal microorganisms or brain neurotransmitters remain to be explored. This study used omics to explore the specific effects of Hp-related chronic gastritis on gastrointestinal microorganisms and brain neurotransmitters in mice, and the specific correlation between gastrointestinal microorganisms and brain neurotransmitters.

## Materials and methods

### Experimental material

A total of 32 SPF grade C57BL/6 mice, half female and half male, 18–22 g, 5–6 weeks old, were supplied by Beijing Weitonglihua Laboratory Animal Technology Co., LTD. Drinking water was filtered bactericidal water, and a drinking water bottle was hung to be freely consumed. The feed and Hp SS1 strain were acquired from Shanghai Puteng Biotechnology Co., LTD. and the Microbial Culture Preservation Center of Guangdong Province, respectively. The Hp SS1 solution was prepared with 0.85% sterile NaCl with a 3 × 10^9^ cfu/mL concentration.

### Model replication

The 32 mice were randomly assigned into a control group (8 males and 8 females) and an experiment group (8 males and 8 females) without significant difference in body weight between the 2 groups as a whole or among the same gender. Both groups gavaged 0.1 mol/L 0.5 mL NaHCO_3_ in the secondary biosafety cabinet to neutralize gastric acid to a weak acid environment suitable for Hp colonization. After 1 h, the experiment group was gavaged with 0.5 mL Hp SS1 bacterial solution of 3 × 10^9^ cfu/mL, and the control group was gavaged with 0.5 mL 0.85% sterile NaCl. It was repeated every 2 days. The intragastric administration was performed 5 times, with 12 h of fasting before each administration. Then, 4 h after the end of each intragastric administration, food and water were fed. The feeding was normal for 12 weeks after the end of the gavage.

### Specimen collection and index detection

After 12 weeks of normal feeding, the mice in the control group and the experimental group were euthanized by cervical dislocation in the biosafety cabinet.

#### Gastric mucosal hp detection and pathological observation

Sterile scissors were used to cut the stomach along the large bend, then lengthwise to an average of two parts. One sample was put into a urease reagent for a urease test to detect Hp. One part was immersed in 10% neutral formalin buffer and fixed immediately, followed by paraffin embedding, sectioning, and HE staining. The new Sydney system (Stolte and Meining, 2001) was used to assess the pathological changes of chronic gastritis, with a score of 0 for none, 1 for slight, 2 for moderate, and 3 for severe. The assessment criteria are specified as follows.

Chronic inflammatory activity was graded according to the degree of neutrophil infiltration: None referred to the absence of neutrophils in the lamina propria; Slight was defined as a small number of neutrophils infiltrating the lamina propria; Moderate referred to the presence of more neutrophils in the mucosal layer, which could be seen in foveolar epithelial cells, surface epithelial cells or glandular epithelium; Severe cases were those in which the neutrophils were relatively dense, or in which a pit abscess might be seen in addition to the moderate ones.

Chronic inflammation was classified according to the density of reactive cells and the depth of infiltration. When the two were inconsistent, the former was mainly used, and the lymphoid follicles and their surrounding small lymphocyte areas must be avoided when calculating: None was defined as mononuclear cells (monocytes, lymphocytes and plasma cells) less than 5 cells per high power field; Slight inflammatory cells were less and limited to the superficial layer of the mucosa, no more than 1/3 of the mucosal layer; Moderate referred to the chronic inflammatory cells which were relatively dense, no more than 2/3 of the mucosal layer; Severe referred to chronic inflammatory cells that were dense and occupied the whole layer of the mucosa.

Atrophy was defined according to the reduction of gastric intrinsic glands: None referred to the lack of obvious reduction in the number of intrinsic glands; Slight was defined as the number of original glands reduced by no more than 1/3; Moderate was defined as a reduction in the number of native glands between 1/3 and 2/3 of the original glands; Severe was defined as the number of intrinsic glands reduced by more than 2/3, only a few glands remained, or even disappeared completely.

Intestinal metaplasia was the replacement of gastric pit, gastric mucosal epithelium and intrinsic gland cells by intestinal type cells (absorptive cells, Panesian cells and/or goblet cells): None referred to the absence of intestinal type cells; Slight was defined as the area of intestinal metaplasia accounting for less than 1/3 of the total area of gland and surface epithelium; Moderate was defined as 1/3 to 2/3 of the total area of the gland and the surface epithelium; Severe was defined as the area of intestinal metaplasia occupying more than 2/3 of the total area of gland and surface epithelium.

Dysplasia was determined according to the morphological changes or tissue structure disorder of gastric mucosal epithelium, gastric pit and intrinsic gland cells: None referred to no abnormal cells; In slight cases, the cells were dense and elongated, with polarity, and the nuclei were mostly located in the basal part with abnormal morphology, and there were few mitotic figures and cell stratification; Moderate was between mild and severe; Severe was defined as dense atypical glands with multilayered nuclei, increased abnormal mitotic figures, and disordered polarity.

#### Gastrointestinal microbial detection

The cecum segment was removed with a sterile scalpel, and the contents were collected. The frozen tube was placed on ice, labeled, placed into liquid nitrogen immediately, and then stored at −80°C. Metagenomics was used to detect gastrointestinal microorganisms, including sample DNA extraction, sample detection, library construction and library detection, up-sequencing, and down-sequencing analysis.

#### Brain neurotransmitter detection

The whole brain tissue sample was separated with sterile surgical instruments. The residual blood of the sample was rinsed with saline solution, and the liquid on the surface of the specimen was dried using filter paper. After rapid freezing, the brain tissue was placed into a frozen tube, marked, and then transferred to a refrigerator at −80°C or liquid nitrogen. Neurotransmitter-targeting metabolomics was used to detect brain neurotransmitters, including standard preparation, sample pretreatment, LC/MS detection, and information analysis.

### Statistical analysis

Measurement data analysis: SPSS 26.0 was utilized for statistical analysis, with a significance level of difference set at *p* < 0.05. Kolmogorov-Smirnov normal distribution test was performed for data rows. Levene’s variance homogeneity test was performed if the normal distribution was satisfied. Two independent samples *t*-test was conducted if the variance was homogeneous. If the variance was not homogeneous, two independent samples *t*-test was conducted. The Wilcoxon rank sum test was performed if it did not conform to the normal distribution.

Metagenomics analysis: Firstly, KneadData software was utilized for quality control (based on Trimmomatic) and de-hosting (based on Bowtie2) of raw data. Before and after KneadData, FastQC was conducted to test the rationality and effect of quality control. Secondly, the microbial database (bacteria, archaea, fungi, viruses) downloaded from Kraken’s website was merged with the microbial database from Kraken2 and the new bacterial genome data from the Columbia University laboratory. The integrated database was used to identify the species contained in the samples, and then Bracken was used to predict the actual relative abundance of species in the samples. Thirdly, based on the abundance of the sample species, the differences in species composition were analyzed by Wayne map and LEfSe analysis.

Targeted metabolomics analysis: First of all, Unsupervised Principal Component Analysis was used for sample data quality control and batch correction. Secondly, the standardization of sample data was carried out in three steps: In-sample correction, that was, the abundance of all features of the sample divided by the median abundance of the sample (similar to relative abundance calculation); Content matrix correction, that was, log transformation of all content values, so that metabolite content distribution was close to normal distribution; Within feature correction, that was, the abundance of all samples corresponding to the feature subtracted the mean abundance of the feature and then divided the standard deviation of the feature abundance, so that the mean and standard deviation of all metabolites were at the same level. Finally, the characteristic metabolites were screened by orthogonal partial least square discriminant analysis (OPLSDA) and random forest, and then the characteristic metabolites were tested by *t*-test.

Pearson correlation analysis was performed between brain neurotransmitters and gastrointestinal microorganisms with significant differences. The results were shown by heat map.

## Results

### Model evaluation

**FIGURE 1:**
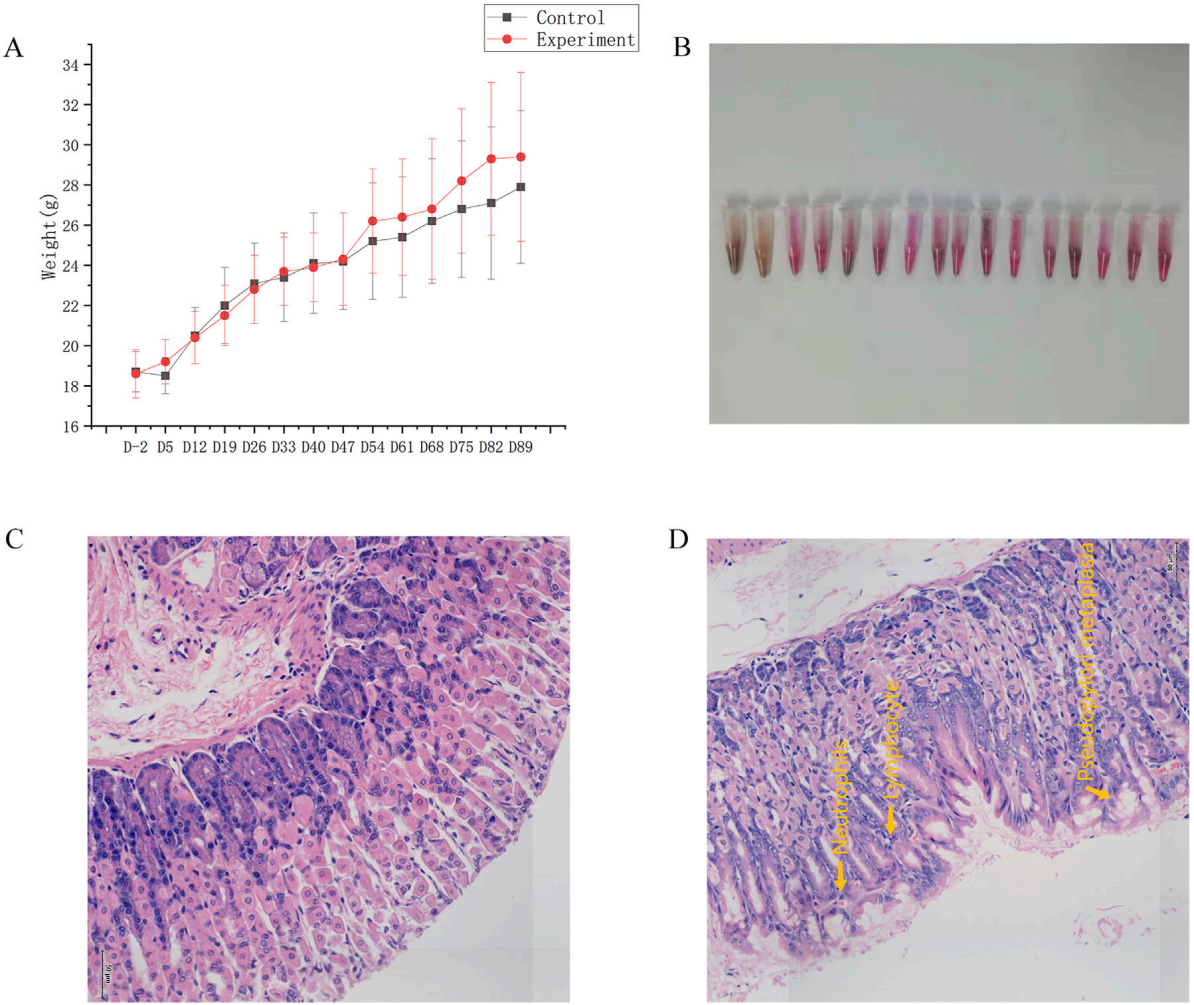
General condition. **(A)** Changes in body weight. **(B)** Results of gastric mucosal urease test to detect Hp in the experiment group. **(C)** Map of gastric mucosa stained with HE in the control group. **(D)** Map of gastric mucosa stained with HE in the experiment group.

Compared to the control group, the hair of the mice in the experiment group was messy, dry, and dull. Figure 1A displayed the weekly measurements of body weight. No notable disparity in body weight was observed between the two groups of mice 2 days before the experiment began (D-2), at the end of the experiment (D89), or at any point during the experiment (*p >* 0.05).

After 12 weeks of modeling, the strong positive rate of gastric mucosal Hp was 87.5% (Figure 1B). In the experiment group, gastric mucosal lymphocytes were infiltrated with local erosion and necrosis, capillary congestion, edema, and exudation. Besides, gastric mucosal folds decreased, and inherent glands decreased and atrophied (Figure 1D). Significant disparities in inflammatory activity, chronic inflammation, and gastric mucosa atrophy were evident in Table 1 when comparing the control group and experiment group as a whole, as well as mice of the same gender (*p* < 0.05). However, there were no notable disparities in gastric mucosa inflammation activity, chronic inflammation, atrophy, intestinal metaplasia, and dysplasia in female and male mice (*p* > 0.05). In conclusion, this experiment successfully replicated the mouse model of Hp-related chronic gastritis, and the study was comparable.

**TABLE 1:** Comparison of gastric mucosal pathological scores $\left(\bar{X}\pm S\right)$ .

	Group	Number of mice	Gastric mucosal pathological score				
			Inflammation activity	Chronic inflammation	Atrophy	Intestinal metaplasia	Dysplasia
1	Control group	16	0.1 ± 0.3	0.1 ± 0.3	0.1 ± 0.3	0.0 ± 0.0	0.0 ± 0.0
	Experiment group	16	1.1 ± 0.3***	1.6 ± 0.5***	0.8 ± 0.4***	0.2 ± 0.4	0.1 ± 0.3
2	Male control group	8	0.1 ± 0.4	0.1 ± 0.4	0.1 ± 0.4	0.0 ± 0.0	0.0 ± 0.0
	Male experiment group	8	1.0 ± 0.0***	1.6 ± 0.5***	0.8 ± 0.5**	0.1 ± 0.4	0.1 ± 0.4
3	Female control group	8	0.1 ± 0.4	0.1 ± 0.4	0.0 ± 0.0	0.0 ± 0.0	0.0 ± 0.0
	Female experiment group	8	1.3 ± 0.5***	1.5 ± 0.5***	0.8 ± 0.5**	0.3 ± 0.5	0.0 ± 0.0
4	Male control group	8	0.1 ± 0.4	0.1 ± 0.4	0.1 ± 0.4	0.0 ± 0.0	0.0 ± 0.0
	Female control group	8	0.1 ± 0.4	0.1 ± 0.4	0.0 ± 0.0	0.0 ± 0.0	0.0 ± 0.0
5	Male experiment group	8	1.0 ± 0.0	1.6 ± 0.5	0.8 ± 0.5	0.1 ± 0.4	0.1 ± 0.4
	Female experiment group	8	1.3 ± 0.5	1.5 ± 0.5	0.8 ± 0.5	0.3 ± 0.5	0.0 ± 0.0

Note: The 5 combinations in the table were respectively examined by pathological score *t*-test, with ** indicating *p* < 0.01, *** indicating *p* < 0.001.

### Comparison of gastrointestinal microorganisms

#### Comparison between the experiment and control groups

**FIGURE 2:**
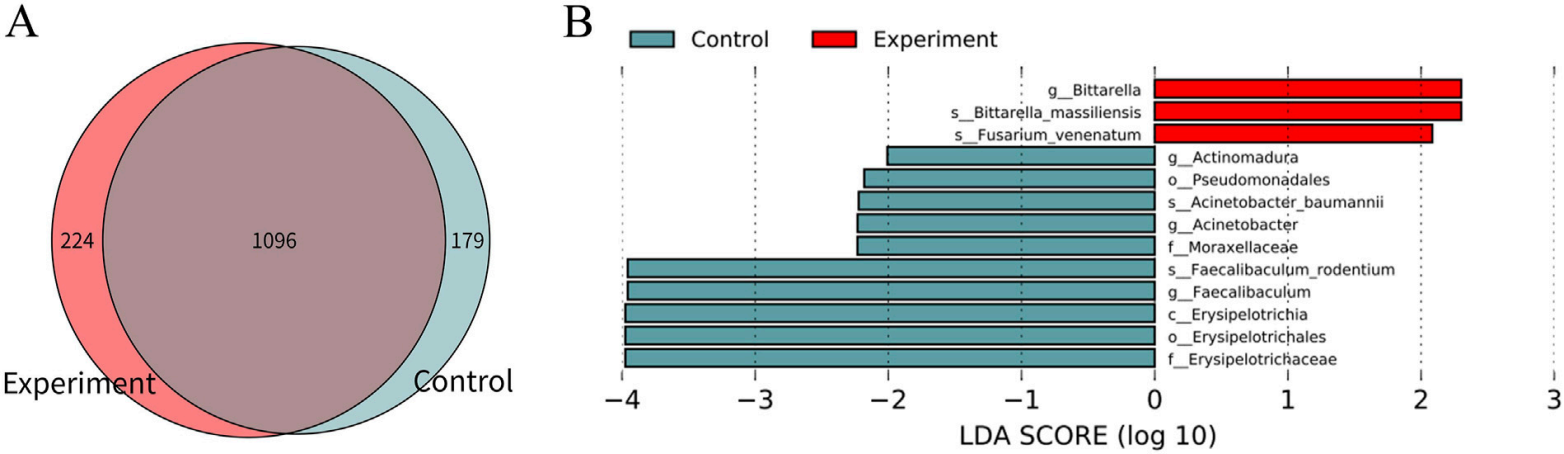
Difference analysis of gastrointestinal microorganisms between the experiment and control groups. **(A)** Wayne map of common and endemic species. **(B)** LDA histogram of LEfSe analysis. C denotes class, o denotes order, f denotes family, g denotes genus, and s denotes species. In the experiment group, 8 cecal colon contents were obtained from male mice and 8 cecal colon contents from female mice. In the control group, 7 cecal colon contents were obtained from male mice and 7 cecal colon contents from female mice, because the sample sizes of one male and one female mouse in the control group was too little to be subjected to metagenomic detection.

There were 1,096 species of gastrointestinal microorganisms shared by the experiment and control groups, including 224 species endemic to the experiment group and 179 species endemic to the control group (Figure 2A). As illustrated in Figure 2B, the two groups had relatively high abundance and significant differences in characteristics of gastrointestinal microorganisms (LDA >2, *p* < 0.05).

#### Comparison between the same gender in the experiment and control groups

**FIGURE 3:**
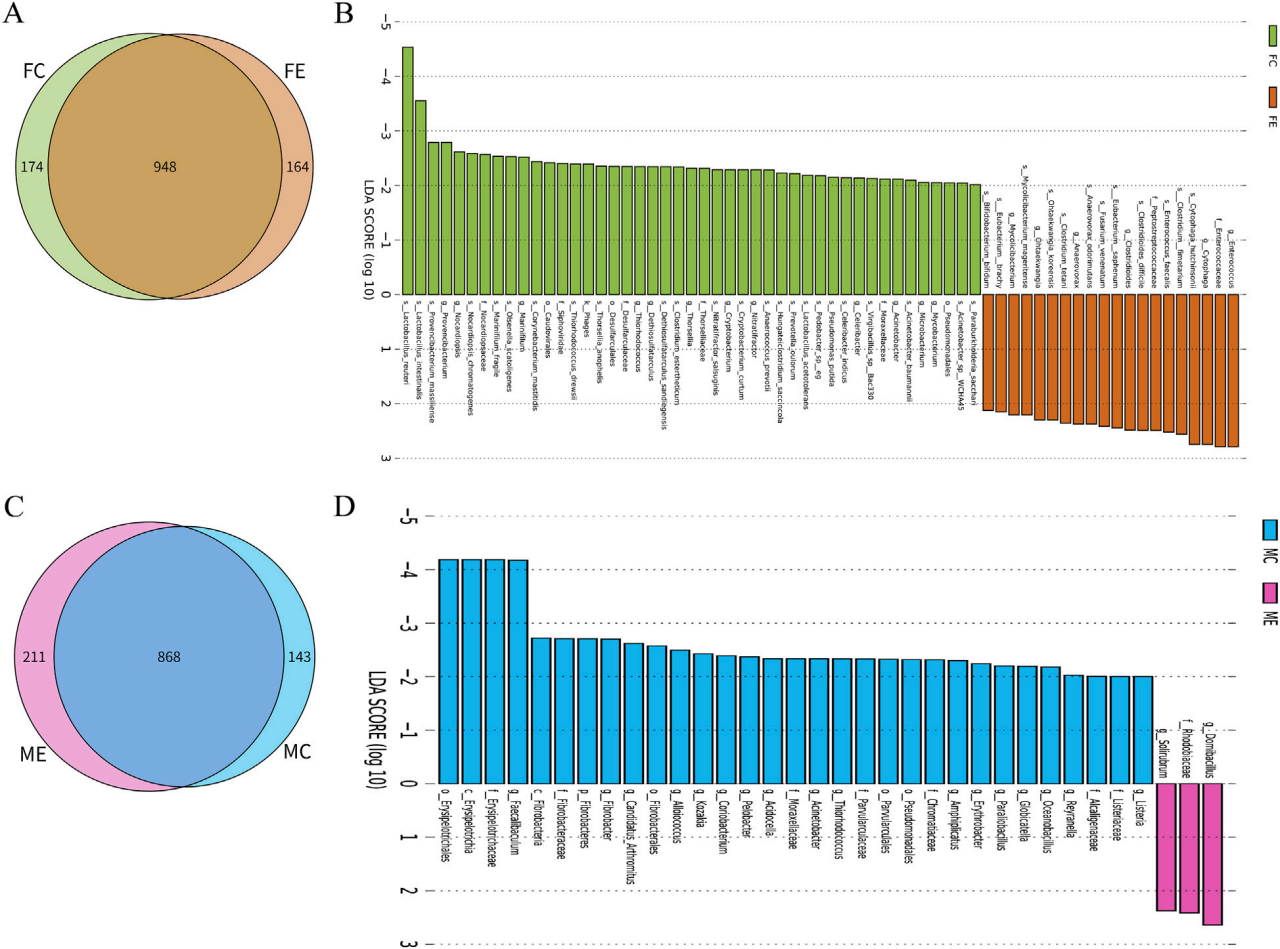
Difference analysis of gastrointestinal microorganisms between the same gender in the experiment and control groups. **(A)** Wayne map of common and endemic species of gastrointestinal microorganisms of female mice in the experiment and control groups. **(B)** LDA histogram of gastrointestinal microbial LEfSe analysis of female mice in the experiment and control groups. **(C)** Wayne map of common and endemic species of gastrointestinal microorganisms of male mice in the experiment and control groups. **(D)** LDA histogram of gastrointestinal microbial LEfSe analysis of male mice in the experiment and control groups. K denotes kingdom, p denotes phylum, c denotes class, o denotes order, f denotes family, g denotes genus, and s denotes species. In the experiment group, 8 cecal colon contents were obtained from male mice and 8 cecal colon contents from female mice. In the control group, 7 cecal colon contents were obtained from male mice and 7 cecal colon contents from female mice.

There were 948 species of gastrointestinal microorganisms shared by the female mice in the experiment and control groups, with 164 and 174 unique species, respectively (Figure 3A). The two groups had their own characteristic microorganisms (LDA >2, *p* < 0.05), as shown in Figure 3B and Supplementary Table S1. There were 868 species of gastrointestinal microorganisms shared by the male experiment group and the male control group, with 211 and 143 unique species, respectively (Figure 3C). The two groups had their own characteristic microorganisms (LDA >2, *p* < 0.05), as shown in Figure 3D and Supplementary Table S2.

#### Comparison between different genders in the experiment and control groups

**FIGURE 4:**
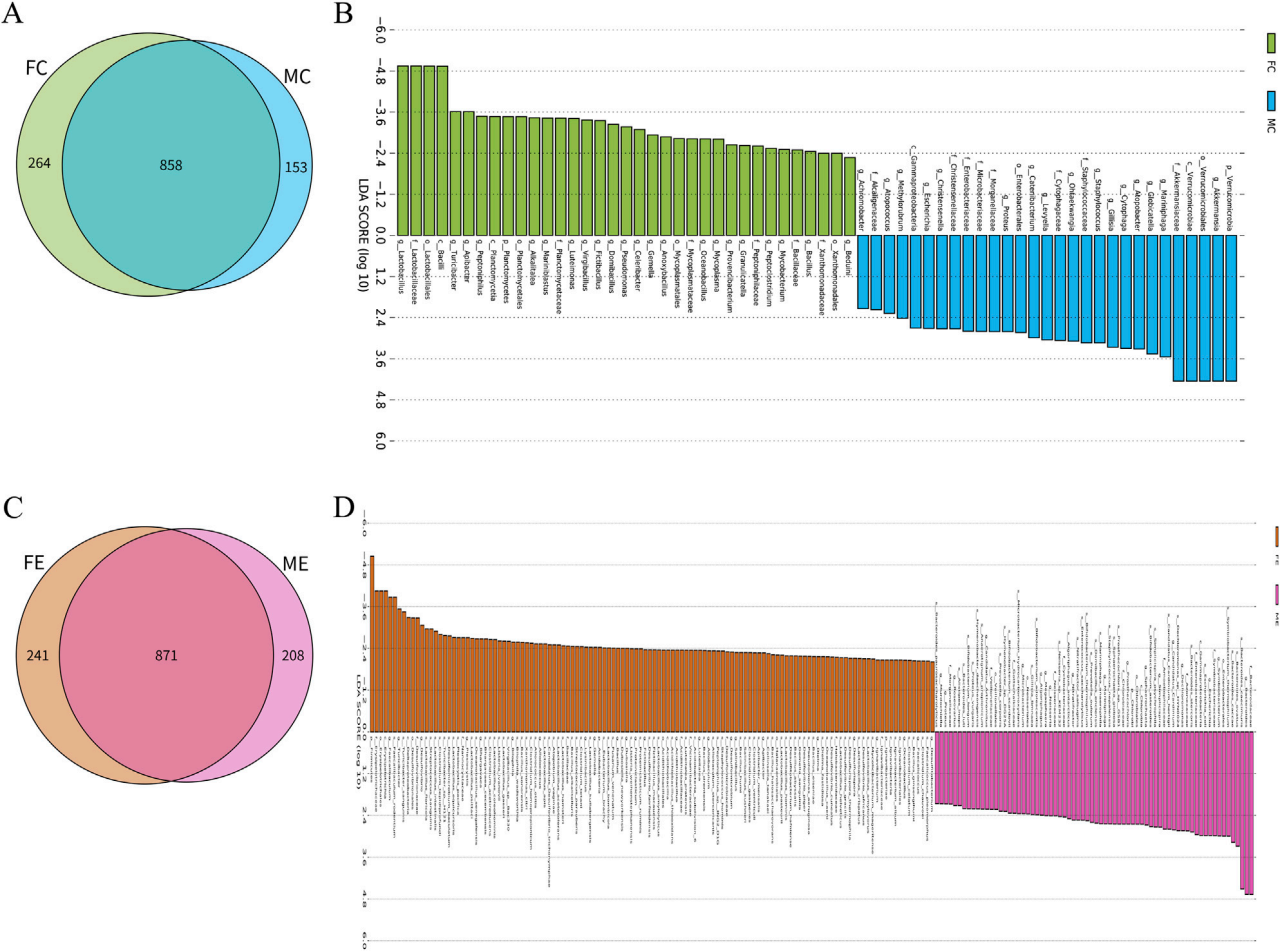
Difference analysis of gastrointestinal microorganisms between different genders in the experiment and control groups. **(A)** Wayne map of common and endemic species of gastrointestinal microorganisms of male and female mice in the control group. **(B)** LDA histogram of gastrointestinal microbial LEfSe analysis of male and female mice in the control group. **(C)** Wayne map of common and endemic species of gastrointestinal microorganisms of male and female mice in the experiment group. **(D)** LDA histogram of gastrointestinal microbial LEfSe analysis of female and male mice in the experiment group. p denotes phylum, c denotes class, o denotes order, f denotes family, g denotes genus, and s denotes species. In the experiment group, 8 cecal colon contents were obtained from male mice and 8 cecal colon contents from female mice. In the control group, 7 cecal colon contents were obtained from male mice and 7 cecal colon contents from female mice.

There were 858 species of gastrointestinal microorganisms shared by male and female mice in the control group, and 153 and 264 species were endemic species, respectively (Figure 4A). Besides, both male and female mice had their own gastrointestinal characteristic microorganisms (LDA >2, *p* < 0.05), as shown in Figure 4B and Supplementary Table S3. In the experiment group, 871 species of gastrointestinal microorganisms were shared by both male and female mice, and 208 and 241 were endemic species, respectively (Figure 4C). Male and female mice had their own gastrointestinal characteristic microorganisms (LDA >2, *p* < 0.05), as shown in Figure 4D and Supplementary Table S4.

### Comparison of brain neurotransmitters

#### Comparison between the experiment and control groups

**FIGURE 5:**
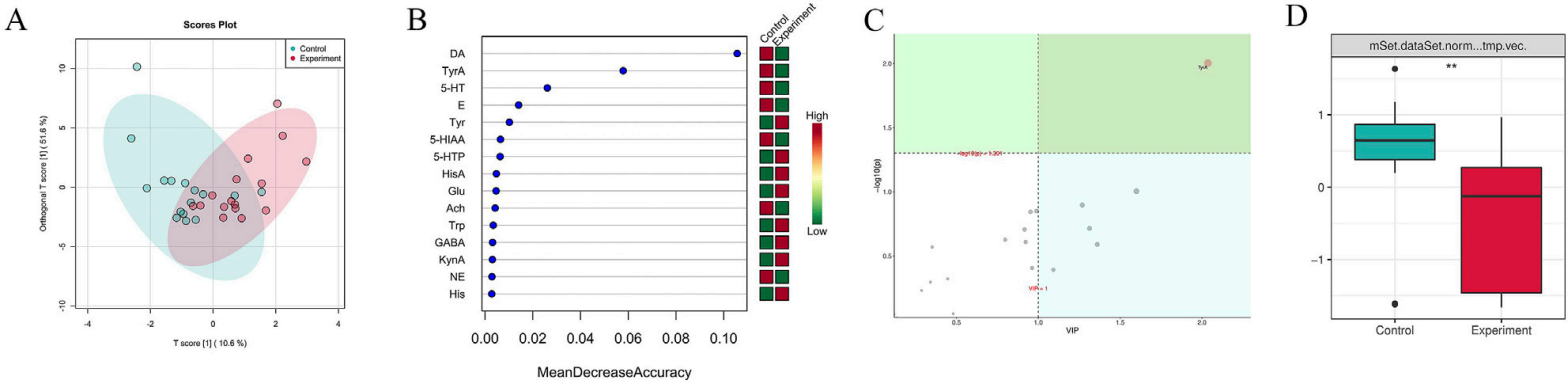
Difference analysis of brain neurotransmitters between the experiment and control groups. **(A)** OPLSDA point cloud diagram. It indicated the overall distribution of the samples. **(B)** Random forest diagram. The horizontal axis of the left panel measured the importance of a metabolite in the random forest, and the right panel was a heat map of the content of 15 metabolites in the two groups. **(C)** OPLSDA metabolite importance diagram. Metabolites with their names marked in yellow were brain neurotransmitters with corrected *p* < 0.05 and VIP >1, which were significantly different between groups. **(D)** Box diagram of brain neurotransmitter TyrA (** denotes *p* < 0.01). It displayed the differences between groups visually. The experiment and control groups obtained 8 brain tissue samples from male mice and 8 brain tissue samples from female mice, respectively.

Figure 5A indicated that there were certain differences in the content of brain neurotransmitters between the experiment and control groups, as observed in the distribution of OPLSDA point clouds. Among the 15 brain neurotransmitters shown in Figure 5B, L-Tyrosine (Tyr), 5-Hydroxytryptophan (5-HTP), Histamine (HisA), L-Glutamic acid (Glu), L-Tryptophan (Trp), 4-Aminobutyric acid (GABA), Kynurenic acid (KynA), and L-Histidine (His) in the experimental group exhibited higher levels compared to the control group, nevertheless the levels of Hydroxytyramine hydrochloride (DA), Tyramine (TyrA), Serotonin hydrochloride (5-HT), Adrenaline hydrochloride (E), 5-Hydroxyindole-3-acetic acid (5-HIAA), Acetylcholine chloride (Ach), and Noradrenaline hydrochloride (NE) in the experiment group were found to be lower compared to the control group. The importance analysis of OPLSDA metabolites in Figure 5C suggested that TyrA content significantly differed between the two groups (VIP >1 and corrected *p* < 0.05). Figure 5D showed that the content of TyrA in the experiment group was considerably lower than that in the control group (*p* < 0.01).

#### Comparison between the same gender in the experiment and control groups

**FIGURE 6:**
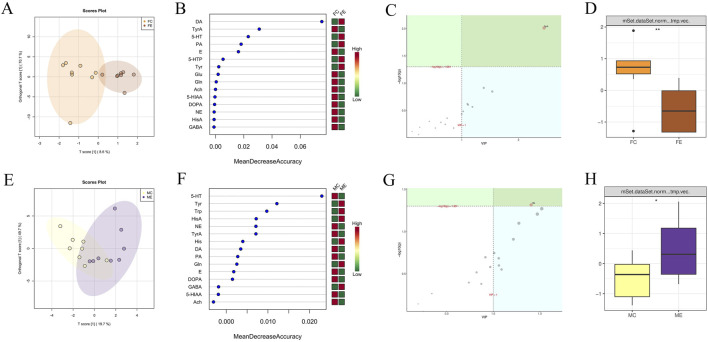
Difference analysis of brain neurotransmitters between the same gender in the experiment and control groups. **(A)** OPLSDA point cloud diagram of brain neurotransmitters of female mice in the experiment and control groups. **(B)** Random forest diagram of brain neurotransmitters of female mice in the experiment and control groups. **(C)** OPLSDA metabolite importance diagram of brain neurotransmitters of female mice in experiment and control groups. **(D)** Box diagram of brain neurotransmitter TyrA of female mice in the experiment and control groups (** indicates *p* < 0.01). **(E)** OPLSDA point cloud diagram of brain neurotransmitters of male mice in experiment and control groups. **(F)** Random forest diagram of brain neurotransmitters of male mice in experiment and control groups. **(G)** OPLSDA metabolite importance diagram of brain neurotransmitters of male mice in experiment and control groups. **(H)** Box diagram of brain neurotransmitter His of male mice in the experiment and control groups (* represents *p* < 0.05). The experiment and control groups obtained 8 brain tissue samples from male mice and 8 brain tissue samples from female mice, respectively.

The OPLSDA point cloud distribution of female mice in the experiment and control groups suggested certain differences (Figure 6A). In the female experimental group, the levels of DA, 5-HT, Picolinic acid (PA), 5-HTP, and Tyr were elevated compared to the female control group among the 15 brain neurotransmitters depicted in Figure 6B. Besides, the levels of brain neurotransmitters in the female experimental group, which included TyrA, E, Glu, L-Glutamine (Gln), Ach, 5-HIAA, Levodopa (DOPA), NE, HisA, and GABA, were found to be decreased compared to those in the female control group. The importance analysis of OPLSDA metabolites in Figure 6C suggested that the TyrA content significantly differed between the two groups (VIP >1 and corrected *p* < 0.05). In Figure 6D, it was found that the amount of TyrA in the female experimental group was considerably reduced compared to the female control group (*p* < 0.01).

The distribution of OPLSDA point clouds of male mice in the experiment and control groups suggested some differences (Figure 6E). Among the 15 brain neurotransmitters shown in Figure 6F, Tyr, Trp, HisA, His, Gln, and GABA in the male experiment group exhibited higher compared to the male control group. The brain neurotransmitters of the male experiment group were lower than that of the male control group, including 5-HT, NE, TyrA, DA, PA, E, DOPA, 5-HIAA, and Ach. The importance analysis of OPLSDA metabolites in Figure 6G indicated that His content significantly differed between the two groups (VIP >1 and corrected *p* < 0.05). In Figure 6H, the His content in the male experiment group exhibited notablely higher than that in the male control group (*p* < 0.05).

#### Comparison between different genders in the experiment and control groups

**FIGURE 7:**
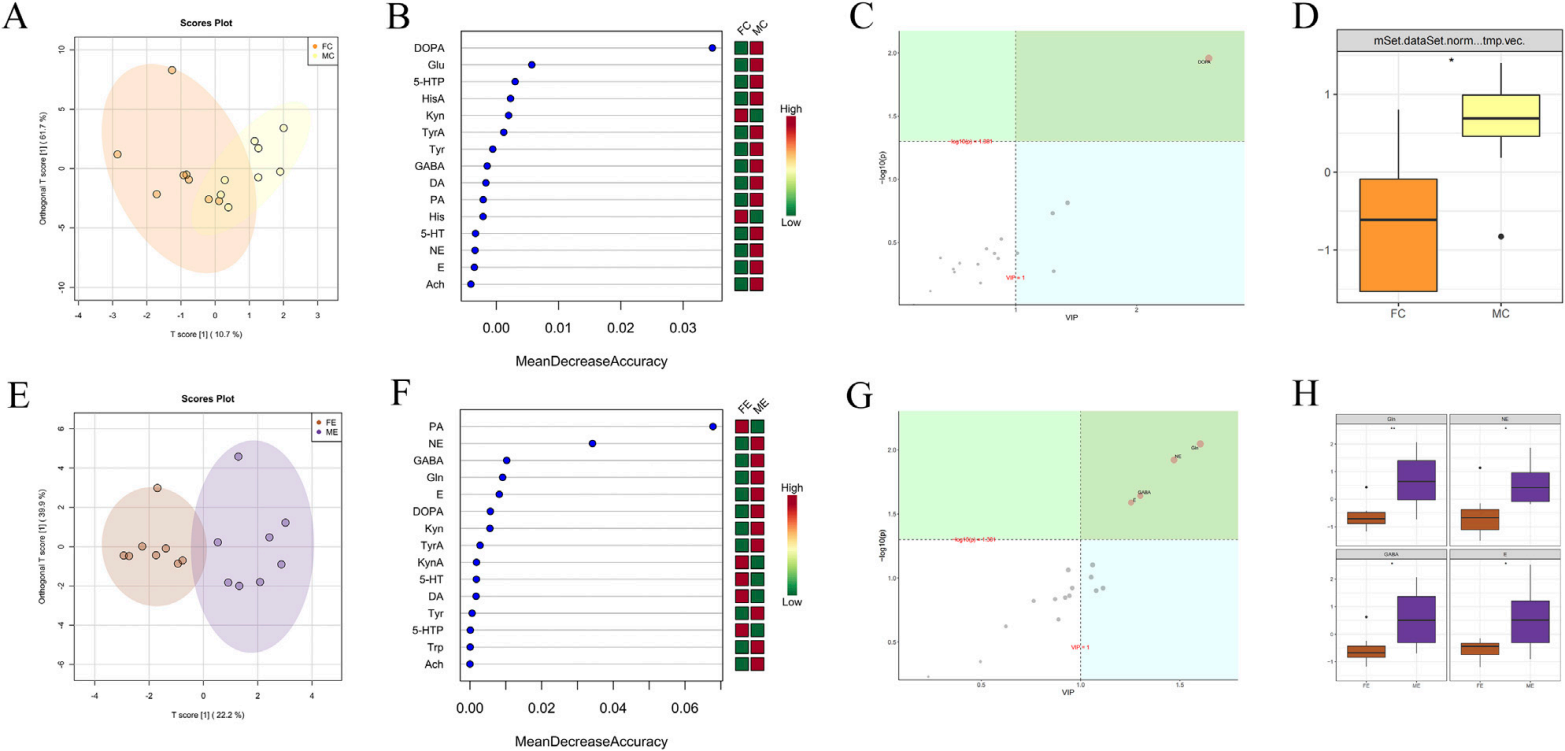
Difference analysis of brain neurotransmitters between different genders in the experiment and control groups. **(A)** OPLSDA point cloud diagram of brain neurotransmitters of male and female mice in the control group. **(B)** Random forest diagram of brain neurotransmitters of male and female mice in the control group. **(C)** OPLSDA metabolite importance diagram of brain neurotransmitters of male and female mice in the control group. **(D)** Box diagram of brain neurotransmitter DOPA of male and female mice in the control group (* represents *p* < 0.05). **(E)** OPLSDA point cloud diagram of brain neurotransmitters of male and female mice in experiment group. **(F)** Random forest diagram of gastrointestinal microorganisms of male and female mice in the experiment group. **(G)** OPLSDA metabolite importance diagram of brain neurotransmitters of male and female mice in the experiment group. **(H)** Box diagram of brain neurotransmitter Gln, NE, GABA, and E of male and female mice in the experiment group (* represents *p* < 0.05, ** indicates *p* < 0.01). The experiment and control groups obtained 8 brain tissue samples from male mice and 8 brain tissue samples from female mice, respectively.

OPLSDA point cloud distribution of male and female mice in the control group suggested some differences in brain neurotransmitter content (Figure 7A). Among the 15 brain neurotransmitters shown in Figure 7B, DL-Kynurenine (Kyn) and His were higher in females than males, and DOPA, Glu, 5-HTP, HisA, TyrA, Tyr, GABA, DA, PA, 5-HT, NE, E, and Ach were lower in females than males. OPLSDA analysis in Figure 7C indicated significant differences in DOPA content between the two groups (VIP >1 and corrected *p* < 0.05), and DOPA content in females was observably lower than that in males (*p* < 0.05).

OPLSDA point cloud distribution of male and female mice in the experiment group suggested some differences in brain neurotransmitter content (Figure 7E). Among the 15 brain neurotransmitters shown in Figure 7F, females had higher levels of PA, KynA, 5-HT, DA, and 5-HTP, while males had higher levels of NE, GABA, Gln, E, DOPA, Kyn, TyrA, Tyr, Trp, and Ach. OPLSDA analysis in Figure 7G indicated that the contents of Gln, NE, GABA, and E significantly differed between the two groups (VIP >1 and corrected *p* < 0.05). The contents of Gln, NE, GABA, and E in females were significantly lower than those in males (*p* < 0.05).

### Correlation analysis between gastrointestinal microorganisms and brain neurotransmitters

The association between gastrointestinal microorganisms with significant differences and brain neurotransmitters was analyzed as shown in the follow figures.

**FIGURE 8:**
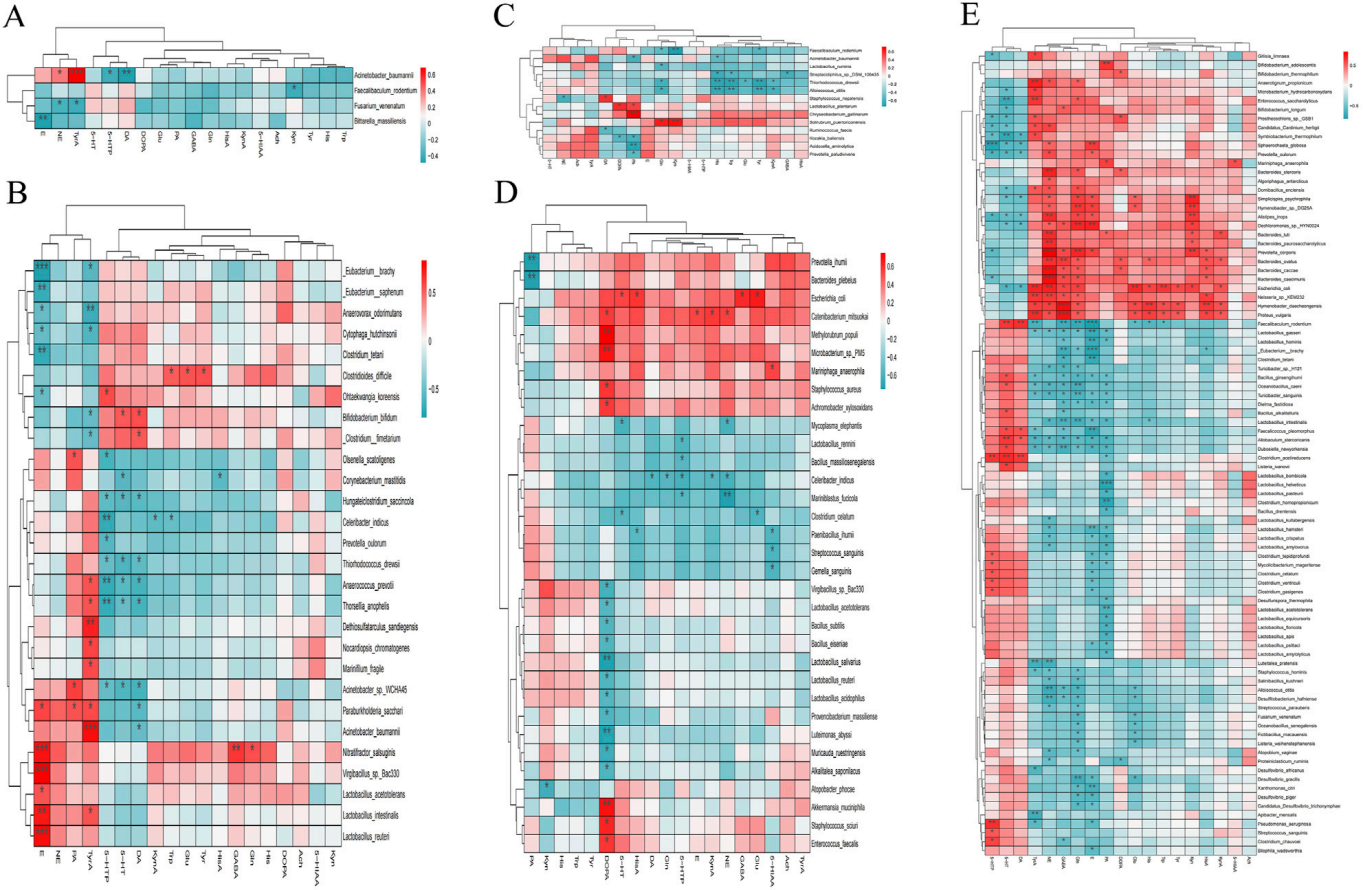
Heat map of the association between brain neurotransmitters and gastrointestinal microorganisms with significant differences. **(A)** Heat map of the association in the experiment and control groups. **(B)** Heat map of the association in the female experiment and control groups. **(C)** Heat map of the association in the male experiment and control groups. **(D)** Heat map of the association in the male and female control groups. **(E)** Heat map of the association in the male and female experiment groups.

The brain TyrA of the experiment and control groups was significantly positively correlated with *Acinetobacter_baumannii* and negatively correlated with *Fusarium_venenatum* (*p* < 0.05; Figure 8A). The brain TyrA of the female experiment group was significantly positively correlated with 8 gastrointestinal microorganisms, such as *Acinetobacter_baumannii*, and negatively correlated with 5 gastrointestinal microorganisms, such as *Anaerovorax_odorimutans* (*p* < 0.05; Figure 8B). The gastrointestinal microorganisms of male mice in the experiment and control groups had no significant positive correlation with the brain His. However, the brain His was significantly negatively correlated with 4 gastrointestinal microbes, including *Acinetobacter_baumannii* (*p* < 0.05; Figure 8C). In comparison between the experimental group and the control group as a whole and between the same gender (Figures 2, 3, 5, 6, 8A–C; Supplementary Tables S1, S2), the brain neurotransmitters with significant differences were significantly correlated with some gastrointestinal microorganisms with significant differences respectively.

Combined with Figures 4, 7, 8D, and Supplementary Tables S3, S4, the correlation between brain neurotransmitters (Gln, GABA, NE, and E) and gastrointestinal microorganisms was comprehensively analyzed as follows.

16 kinds of gastrointestinal microorganisms like *Alistipes_inops*, which were significantly positively correlated with brain Gln, were significantly lower in female mice with Hp-related chronic gastritis than in male mice (LDA >2 and *p* < 0.05). However, among the 16 gastrointestinal microorganisms, only *Escherichia_coli* in female normal mice was significantly lower than in male normal mice (LDA >2 and *p* < 0.05). Furthermore, 25 kinds of gastrointestinal microorganisms, which were significantly negatively correlated with brain Gln, such as *Allobaculum_stercoricanis*, were significantly more abundant in female mice with Hp-related chronic gastritis than in male mice (LDA >2 and *p* < 0.05). Among the 25 kinds of gastrointestinal microorganisms, only 9 kinds of gastrointestinal microbial abundance in female normal mice, such as *Bacillus_ginsengihumi*, were also significantly higher than that of male normal mice (LDA >2 and *p* < 0.05).

The abundances of 10 kinds of gastrointestinal microorganisms, such as *Bacteroides_caccae*, with a significant positive correlation of GABA, in the female mice with Hp-related chronic gastritis, were significantly lower than those in the male mice (LDA >2 and *p* < 0.05). Among the 10 kinds of gastrointestinal microorganisms, only *Escherichia_coli* abundance in female normal mice was significantly lower than that in male normal mice (LDA >2 and *p* < 0.05). The abundance of 18 gastrointestinal microorganisms, which had a significant negative correlation with brain GABA, such as *Allobaculum_stercoricanis*, in female mice with Hp-related chronic gastritis, were significantly higher than those in male mice (LDA >2 and *p* < 0.05). Among which only 8 kinds of gastrointestinal microorganisms such as *Bacillus_alkalitelluris* in female normal mice were also significantly higher than those in male normal mice (LDA >2 and *p* < 0.05).

The abundance of 21 gastrointestinal microorganisms, such as *Algoriphagus_antarcticus*, which had a significant positive correlation with brain NE, were significantly lower in female mice than in male mice with Hp-related chronic gastritis (LDA >2 and *p* < 0.05). Among the 21 kinds of gastrointestinal microorganisms, only *Escherichia_coli* abundance in female normal mice was significantly lower than in male normal mice (LDA >2 and *p* < 0.05). The abundance of 19 gastrointestinal microorganisms, such as *Allobaculum_stercoricanis*, which had a significant negative correlation with brain NE, were significantly higher in female mice than in male mice with Hp-related chronic gastritis (LDA >2 and *p* < 0.05). Among the 19 kinds of gastrointestinal microorganisms, the abundance of 9 gastrointestinal microorganisms, including *Lactobacillus_amylovorus*, were significantly higher in female normal mice than in male normal mice (LDA >2 and *p* < 0.05).

The abundance of 7 gastrointestinal microorganisms like *Alistipes_inops*, which were significantly positively correlated with brain E, were significantly lower in female mice than in male mice with Hp-related chronic gastritis (LDA >2 and *p* < 0.05). Among the 7 kinds of gastrointestinal microorganisms, there were no significant difference between male and female normal mice (LDA ≤2 or *p* > 0.05). The abundance of 26 gastrointestinal microorganisms, such as *Allobaculum_stercoricanis*, which had a significant negative correlation with brain E, were significantly higher in female mice than those in male mice with Hp-related chronic gastritis (LDA >2 and *p* < 0.05), of which only 8 kinds of gastrointestinal microbial abundance such as *Bacillus_ginsengihumi* in female normal mice also significantly higher than those of male normal mice (LDA >2 and *p* < 0.05).

Among the gastrointestinal microorganisms significantly related to Gln, GABA, NE, and E, only a few showed the same significant differences of male and female mice between the experiment group and the control group, indicating that Hp-related chronic gastritis caused new differences in gastrointestinal microorganisms between male and female mice to a certain extent.

## Discussion

Chronic gastritis is an inflammatory disease of gastric mucosa (Solovyova et al., 2019). The incidence of chronic gastritis is closely related to Hp (Malfertheiner et al., 2023; Zhu et al., 2022), and Hp-related chronic gastritis severely affects human health (Alizadeh-Naini et al., 2020; Mestrovic et al., 2021).

Hp-related chronic gastritis model replication methods have been mature, and C57BL/6 mice are relatively easy to colonize by Hp (Dey et al., 2021; Forooghi et al., 2023; Malespín-Bendaña et al., 2023). In this research, C57BL/6 mice were selected as experimental subjects, and the Hp infection method was used to replicate the model of Hp-related chronic gastritis. In the experiment group, the strong positive rate of Hp in the gastric mucosa of mice was 87.5%, and all mice had pathological changes to different degrees of chronic gastritis, indicating that the mouse model was successfully replicated in this experiment. Significant differences were found in inflammatory activity, chronic inflammation, and gastric mucosa atrophy between the same-sex mice in the experiment and control groups. There were no significant differences in the gastric mucosal pathology of male and female mice in the same group. Therefore, gastrointestinal microorganisms and brain neurotransmitters between and within the experiment and control groups were comparable.

Studies have shown that Hp infection could have a certain impact on gastrointestinal microflora and gastrointestinal diseases (Chen et al., 2022; Pereira-Marques et al., 2019). In this research, C57BL/6 mice were selected as the object, and the differences in gastrointestinal microecology between Hp-related chronic gastritis mice and normal mice have been discussed in detail. There were significant differences in gastrointestinal microorganisms between mice with Hp-related chronic gastritis and normal mice as a whole and the same-sex mice. The specific differences are shown in Figures 2, 3 and Supplementary Tables S1, S2, indicating that Hp-related chronic gastritis caused some significant changes in the gastrointestinal microorganisms of mice. Some specific differences were related to the gender of the mice. There were significant differences between the male and female mice with Hp-related chronic gastritis, as well as between the male and female normal mice, but the differences were not entirely the same, as shown in Figure 4 and Supplementary Tables S3, S4. These indicate that the significant differences in gastrointestinal microorganisms of male and female mice with Hp-related chronic gastritis were not entirely caused by the gender of mice, and Hp-related chronic gastritis also led to certain differences. Some scholars have studied the effect of Hp infection on gastrointestinal microorganisms (Elghannam et al., 2024; Lino and Shimoyama, 2021; Tao et al., 2020), but no special study has been conducted on the effect of chronic gastritis caused by Hp infection on gastrointestinal microorganisms, let alone the difference in gender. This study fills the gap of how experimental Hp-related chronic gastritis specifically affects the gastrointestinal microbiota in mice. It will provide the possibility to explain the effects of Hp-related chronic gastritis on the gastrointestinal tract and other parts from the perspective of gastrointestinal microecology.

There are some evidences that Hp may be closely related to central nervous and peripheral nervous system diseases, but the specific mechanism has been unclear (Baj et al., 2021; Dardiotis et al., 2020; Sticlaru et al., 2019). Neurotransmitters are closely linked to the pathogenesis of nervous system diseases, and numerous studies have been conducted in this area (Özakman et al., 2021; Schützler et al., 2019; Schaeffer et al., 2021). However, the association between Hp and neurotransmitters has been unclear. This study conducted a preliminary exploration on Hp-related chronic gastritis and brain neurotransmitters in mice. These results indicated that Hp-related chronic gastritis might cause significant changes in mice’s brain neurotransmitters, and the specific differences varied based on gender. The significant differences in brain Gln, GABA, NE, E contents and the disappearance of significant difference in brain DOPA content in male and female mice with Hp-related chronic gastritis were not the differences between male and female normal mice, but the changes might be caused by Hp-related chronic gastritis. The exact effect of Hp-related chronic gastritis on brain neurotransmitters will provide experimental reference for the study of the mechanism of Hp-related chronic gastritis on nervous system diseases and symptoms.

With the advancement of research on the microbiota-gut-brain axis, increasing evidences indicate a close relationship between microorganisms and neurotransmitters (Strandwitz, 2018; Gorlé et al., 2021; Dicks, 2022). This study analyzed the relationship of gastrointestinal microorganisms and brain neurotransmitters between the mice with Hp-related chronic gastritis and normal mice (Figure 8), and revealed some correlations between brain neurotransmitters and gastrointestinal microorganisms. The results delve into a possible phenomenon that Hp-related chronic gastritis could cause the differences of gastrointestinal microorganisms and brain neurotransmitters, and the changes of brain neurotransmitters might be related to the changes in gastrointestinal microorganisms. Many studies have shown that Hp infection may be widely associated with gastric and extragastric diseases (Santos et al., 2020; Idowu et al., 2022). This study focused on the effects of Hp-related chronic gastritis on gastrointestinal microorganisms and brain neurotransmitters in mice, providing an experimental basis for the research on the association mechanism.

This experiment initially revealed the specific effects of experimental Hp-related chronic gastritis on gastrointestinal microorganisms and brain neurotransmitters in mice, and the possible relationship between gastrointestinal microorganisms and brain neurotransmitters. However, related research involves multi-factors, multi-links and multi-stages. For example, the specific influence mechanism of Hp-related chronic gastritis on gastrointestinal microecology, the specific influence pathway between gastrointestinal microecology and brain neurotransmitters, the association between Hp-related chronic gastritis and nervous system diseases, and the gender difference mechanism of Hp-related chronic gastritis model mice revealed by this experiment remain to be further explored. In the future, further studies will be conducted based on the results of this study.

## Conclusion

Hp-related chronic gastritis has certain effects on gastrointestinal microorganisms and brain neurotransmitters in mice, and the specific effects differ to certain degrees depending on gender. In addition, the changes in brain neurotransmitters in mice with Hp-related chronic gastritis may be related to the changes in gastrointestinal microorganisms.

## Data Availability

The original contributions presented in the study are included in the article/Supplementary Material, further inquiries can be directed to the corresponding author.

## References

[B1] Alizadeh-Naini M. Yousefnejad H. Hejazi N. (2020). The beneficial health effects of Nigella sativa on *Helicobacter pylori* eradication, dyspepsia symptoms, and quality of life in infected patients: a pilot study. Phytotherapy Res. PTR 34 (6), 1367–1376. 10.1002/ptr.6610 31916648

[B2] Al Quraan A. M. Beriwal N. Sangay P. Namgyal T. (2019). The psychotic impact of Helicobacter pylori gastritis and functional dyspepsia on depression: a systematic review. Cureus 11 (10), e5956. 10.7759/cureus.5956 31799095 PMC6863582

[B3] Baj J. Forma A. Flieger W. Morawska I. Michalski A. Buszewicz G. (2021). *Helicobacter pylori* infection and extragastric diseases-A focus on the central nervous system. Cells 10 (9), 2191. 10.3390/cells10092191 34571840 PMC8469861

[B4] Chen X. Wang N. Wang J. Liao B. Cheng L. Ren B. (2022). The interactions between oral-gut axis microbiota and *Helicobacter pylori* . Front. Cell. Infect. Microbiol. 12, 914418. 10.3389/fcimb.2022.914418 35992177 PMC9381925

[B5] Dardiotis E. Sokratous M. Tsouris Z. Siokas V. Mentis A. A. Aloizou A. M. (2020). Association between *Helicobacter pylori* infection and guillain-barré syndrome: a meta-analysis. Eur. J. Clin. investigation 50 (5), e13218. 10.1111/eci.13218 32124432

[B6] Dey T. K. Karmakar B. C. Sarkar A. Paul S. Mukhopadhyay A. K. (2021). A mouse model of *Helicobacter pylori* infection. Methods Mol. Biol. (Clifton, N.J.) 2283, 131–151. 10.1007/978-1-0716-1302-3_14 33765316

[B7] Dicks L. M. T. (2022). Gut bacteria and neurotransmitters. Microorganisms 10 (9), 1838. 10.3390/microorganisms10091838 36144440 PMC9504309

[B8] Doulberis M. Kotronis G. Gialamprinou D. Polyzos S. A. Papaefthymiou A. Katsinelos P. (2021). Alzheimer's disease and gastrointestinal microbiota; impact of *Helicobacter pylori* infection involvement. Int. J. Neurosci. 131 (3), 289–301. 10.1080/00207454.2020.1738432 32125206

[B9] Elghannam M. T. Hassanien M. H. Ameen Y. A. Turky E. A. Elattar G. M. Elray A. A. (2024). *Helicobacter pylori* and oral-gut microbiome: clinical implications. Infection 52 (2), 289–300. 10.1007/s15010-023-02115-7 37917397 PMC10954935

[B10] Forooghi Nia F. Rahmati A. Ariamanesh M. Saeidi J. Ghasemi A. Mohtashami M. (2023). The Anti-*Helicobacter pylori* effects of Limosilactobacillus reuteri strain 2892 isolated from Camel milk in C57BL/6 mice. World J. Microbiol. and Biotechnol. 39 (5), 119. 10.1007/s11274-023-03555-x 36918449

[B11] Gorlé N. Bauwens E. Haesebrouck F. Smet A. Vandenbroucke R. E. (2021). Helicobacter and the potential role in neurological disorders: there is more than *Helicobacter pylori* . Front. Immunol. 11, 584165. 10.3389/fimmu.2020.584165 33633723 PMC7901999

[B12] Hanafiah A. Lopes B. S. (2020). Genetic diversity and virulence characteristics of *Helicobacter pylori* isolates in different human ethnic groups. Infect. Genet. Evol. J. Mol. Epidemiol. Evol. Genet. Infect. Dis. 78, 104135. 10.1016/j.meegid.2019.104135 31837482

[B13] He J. Liu Y. Ouyang Q. Li R. Li J. Chen W. (2022). *Helicobacter pylori* and unignorable extragastric diseases: mechanism and implications. Front. Microbiol. 13, 972777. 10.3389/fmicb.2022.972777 35992650 PMC9386483

[B14] Idowu S. Bertrand P. P. Walduck A. K. (2022). Gastric organoids: advancing the study of *H. pylori* pathogenesis and inflammation. Helicobacter 27 (3), e12891. 10.1111/hel.12891 35384141 PMC9287064

[B15] Kalach N. Zrinjka M. Bontems P. Kori M. Homan M. Cabral J. (2022). Systematic review and meta-analysis of histological gastric biopsy aspects according to the updated Sydney system in children. J. Pediatr. gastroenterology Nutr. 74 (1), 13–19. 10.1097/MPG.0000000000003259 34338237

[B16] Lino C. Shimoyama T. (2021). Impact of *Helicobacter pylori* infection on gut microbiota. World J. gastroenterology 27 (37), 6224–6230. 10.3748/wjg.v27.i37.6224 PMC8515792 34712028

[B17] Lopetuso L. R. Scaldaferri F. Franceschi F. Gasbarrini A. (2014). The gastrointestinal microbiome - functional interference between stomach and intestine. Best Pract. and Res. Clin. gastroenterology. 28 (6), 995–1002. 10.1016/j.bpg.2014.10.004 25439066

[B18] Lovino L. Tremblay M. E. Civiero L. (2020). Glutamate-induced excitotoxicity in Parkinson's disease: the role of glial cells. J. Pharmacol. Sci. 144 (3), 151–164. 10.1016/j.jphs.2020.07.011 32807662

[B19] Malespín-Bendaña W. Alpízar-Alpízar W. Figueroa-Protti L. Reyes L. Molina-Castro S. Une C. (2023). *Helicobacter pylori* infection induces gastric precancerous lesions and persistent expression of Angpt2, Vegf-A and Tnf-A in a mouse model. Front. Oncol. 13, 1072802. 10.3389/fonc.2023.1072802 36874142 PMC9975564

[B20] Malfertheiner P. Camargo M. C. El-Omar E. Liou J. M. Peek R. Schulz C. (2023). *Helicobacter pylori* infection. Nat. Rev. Dis. Prim. 9 (1), 19. 10.1038/s41572-023-00431-8 37081005 PMC11558793

[B21] Mestrovic A. Bozic J. Vukojevic K. Tonkic A. (2021). Impact of different *Helicobacter pylori* eradication therapies on gastrointestinal symptoms. Med. Kaunas. Lith. 57 (8), 803. 10.3390/medicina57080803 PMC8400225 34441009

[B22] Nimgampalle M. Chakravarthy H. Sharma S. Shree S. Bhat A. R. Pradeepkiran J. A. (2023). Neurotransmitter systems in the etiology of major neurological disorders: emerging insights and therapeutic implications. Ageing Res. Rev. 89, 101994. 10.1016/j.arr.2023.101994 37385351

[B23] Özakman S. Gören M. Z. Nurten A. Tekin N. Kalaycı R. Enginar N. (2021). Effects of tamoxifen and glutamate and glutamine levels in brain regions in repeated sleep deprivation-induced mania model in mice. Naunyn-Schmiedeberg's archives Pharmacol. 394 (4), 619–629. 10.1007/s00210-020-02001-1 33104849

[B24] Pereira-Marques J. Ferreira R. M. Pinto-Ribeiro I. Figueiredo C. (2019). *Helicobacter pylori* infection, the gastric microbiome and gastric cancer. Adv. Exp. Med. Biol. 1149, 195–210. 10.1007/5584_2019_366 31016631

[B25] Rahman M. O. Islam A. S. Choudhury M. S. Raihan A. A. Alam M. S. Chowdury M. (2020). A study of association between H. Pylori genotype and chronic gastritis. Mymensingh Med. J. MMJ. 29 (3), 664–675. 32844810

[B26] Santos M. L. C. de Brito B. B. da Silva F. A. F. Sampaio M. M. Marques H. S. Oliveira E Silva N. (2020). *Helicobacter pylori* infection: beyond gastric manifestations. World J. gastroenterology 26 (28), 4076–4093. 10.3748/wjg.v26.i28.4076 PMC7403793 32821071

[B27] Schaeffer E. Vaterrodt T. Zaunbrecher L. Liepelt-Scarfone I. Emmert K. Roeben B. (2021). Effects of Levodopa on quality of sleep and nocturnal movements in Parkinson's Disease. J. neurology 268 (7), 2506–2514. 10.1007/s00415-021-10419-7 PMC8216994 33544218

[B28] Schulz C. Schütte K. Koch N. Vilchez-Vargas R. Wos-Oxley M. L. Oxley A. P. A. (2018). The active bacterial assemblages of the upper GI tract in individuals with and without Helicobacter infection. Gut 67 (2), 216–225. 10.1136/gutjnl-2016-312904 27920199

[B29] Schützler N. Girwert C. Hügli I. Mohana G. Roignant J. Y. Ryglewski S. (2019). Tyramine action on motoneuron excitability and adaptable tyramine/octopamine ratios adjust Drosophila locomotion to nutritional state. Proc. Natl. Acad. Sci. U. S. A. 116 (9), 3805–3810. 10.1073/pnas.1813554116 30808766 PMC6397572

[B30] Shen X. Yang H. Wu Y. Zhang D. Jiang H. (2017). Meta-analysis: association of *Helicobacter pylori* infection with Parkinson's diseases. Helicobacter. 22(5). 10.1111/hel.12398 28598012

[B31] Solovyova G. Alianova T. Kuryk O. Taran A. (2019). Morphological peculiarities of chronic gastritis in patients with functional dyspepsia. Georgian Med. news (289), 102–107. 31215888

[B32] Sticlaru L. Stăniceanu F. Cioplea M. Nichita L. Bastian A. Micu G. (2019). Dangerous liaison: *Helicobacter pylori*, ganglionitis, and myenteric gastric neurons: a histopathological study. Anal. Cell. Pathol. Amst. 2019, 3085181. 10.1155/2019/3085181 32082967 PMC7012220

[B33] Stolte M. Meining A. (2001). The updated Sydney system: classification and grading of gastritis as the basis of diagnosis and treatment. Can. J. gastroenterology = J. Can. de gastroenterologie 15 (9), 591–598. 10.1155/2001/367832 11573102

[B34] Strandwitz P. (2018). Neurotransmitter modulation by the gut microbiota. Brain Res. 1693 (Pt B), 128–133. 10.1016/j.brainres.2018.03.015 29903615 PMC6005194

[B35] Sung Y. F. Yin J. H. Lee K. H. Tsai C. L. Lin Y. K. Chen S. Y. (2022). Increased risk of sleep-related movement disorder in patients with *Helicobacter pylori* infection: a nationwide population-based study. Front. neurology 13, 953821. 10.3389/fneur.2022.953821 PMC9589275 36299273

[B36] Suzuki H. Ataka K. Asakawa A. Cheng K. C. Ushikai M. Iwai H. (2019). *Helicobacter pylori* vacuolating cytotoxin A causes anorexia and anxiety via hypothalamic urocortin 1 in mice. Sci. Rep. 9 (1), 6011. 10.1038/s41598-019-42163-4 30979915 PMC6461611

[B37] Tao Z. H. Han J. X. Fang J. Y. (2020). *Helicobacter pylori* infection and eradication: exploring their impacts on the gastrointestinal microbiota. Helicobacter 25 (6), e12754. 10.1111/hel.12754 32876377

[B38] Yin Y. Liang H. Wei N. Zheng Z. (2022). Prevalence of chronic atrophic gastritis worldwide from 2010 to 2020: an updated systematic review and meta-analysis. Ann. Palliat. Med. 11 (12), 3697–3703. 10.21037/apm-21-1464 36635994

[B39] Zhao Y. Gao X. Guo J. Yu D. Xiao Y. Wang H. (2019). *Helicobacter pylori* infection alters gastric and tongue coating microbial communities. Helicobacter 24 (2), e12567. 10.1111/hel.12567 30734438 PMC6593728

[B40] Zhu X. Zhu C. Zhao Y. Liu X. Sa R. Wang Y. (2022). Prevalence of *Helicobacter pylori* virulence genes and their association with chronic gastritis in Beijing, China. Curr. Microbiol. 80 (1), 33. 10.1007/s00284-022-03135-6 36482124

