# Supplementary material for: Effect of Helicobacter pylori-related chronic gastritis on gastrointestinal microorganisms and brain neurotransmitters in mice
Source: Front Pharmacol. 2024 Dec 6;15:1472437. doi: 10.3389/fphar.2024.1472437 (PMC11659015; doi:10.3389/fphar.2024.1472437)
Supplement: Supplementary file 1 [file Table1.DOCX]

**Supplementary Table 1**

Gastrointestinal characteristic microorganisms of female mice in the experiment and control group.

| **Group** | **Gastrointestinal characteristic microorganisms（LDA > 2, *P* < 0.05）** |
| --- | --- |
| Female control group | k__Phages、o__Caudovirales、o__Desulfarculales、o__Pseudomonadales、f__Desulfarculaceae、f__Moraxellaceae、f__Nocardiopsaceae、  f__Siphoviridae、f__Thorselliaceae、g__Acinetobacter、  g__Celeribacter、g__Cryptobacterium、g__Dethiosulfatarculus、  g__Marinifilum、g__Microbacterium、g__Mycobacterium、  g__Nitratifractor、g__Nocardiopsis、g__Provencibacterium、  g__Thiorhodococcus、g__Thorsellia、s__Acinetobacter_baumannii、  s__Acinetobacter_sp__WCHA45、s__Anaerococcus_prevotii、  s__Celeribacter_indicus、s__Clostridium_estertheticum、  s__Corynebacterium_mastitidis、s__Cryptobacterium_curtum、  s__Dethiosulfatarculus_sandiegensis、s__Hungateiclostridium_saccincola、s__Lactobacillus_acetotolerans、s__Lactobacillus_intestinalis、s__Lactobacillus_reuteri、s__Marinifilum_fragile、s__Nitratifractor_salsuginis、  s__Nocardiopsis_chromatogenes、s__Olsenella_scatoligenes、  s__Paraburkholderia_sacchari、s__Pedobacter_sp__eg、  s__Prevotella_oulorum、s__Provencibacterium_massiliense、  s__Pseudomonas_putida、s__Thiorhodococcus_drewsii、  s__Thorsellia_anophelis、s__Virgibacillus_sp__Bac330 |
| Female experiment group | f__Enterococcaceae、f__Peptostreptococcaceae、g__Anaerovorax、  g__Clostridioides、g__Cytophaga、g__Enterococcus、  g__Mycolicibacterium、g__Ohtaekwangia、s___Clostridium__fimetarium、  s___Eubacterium__brachy、s___Eubacterium__saphenum、  s__Anaerovorax_odorimutans、s__Bifidobacterium_bifidum、  s__Clostridioides_difficile、s__Clostridium_tetani、s__Cytophaga_hutchinsonii、s__Enterococcus_faecalis、s__Fusarium_venenatum、s__Mycolicibacterium_mageritense、s__Ohtaekwangia_koreensis |

Note: P denotes phylum, c denotes class, o denotes order, f denotes family, g denotes genus, and s denotes species.
