# Supplementary material for: Effect of Helicobacter pylori-related chronic gastritis on gastrointestinal microorganisms and brain neurotransmitters in mice
Source: Front Pharmacol. 2024 Dec 6;15:1472437. doi: 10.3389/fphar.2024.1472437 (PMC11659015; doi:10.3389/fphar.2024.1472437)
Supplement: Supplementary file 2 [file Table2.DOCX]

**Supplementary Table 2**

Gastrointestinal characteristic microorganisms of male mice in the experiment and control group.

| **Group** | | **Gastrointestinal characteristic microorganisms（LDA > 2, *P* < 0.05）** |
| --- | --- | --- |
| Male control group | p__Fibrobacteres、c__Erysipelotrichia、c__Fibrobacteria、o__Erysipelotrichales、o__Fibrobacterales、o__Pseudomonadales、f__Alcaligenaceae、f__Chromatiaceae、f__Erysipelotrichaceae、f__Fibrobacteraceae、f__Moraxellaceae、g__Acidocella、  g__Acinetobacter、g__Alloiococcus、g__Candidatus_Arthromitus、  g__Dubosiella、g__Faecalibaculum、g__Fibrobacter、g__Kozakia、  g__Oceanobacillus、g__Thiorhodococcus、s__Acidocella_aminolytica、  s__Acinetobacter_baumannii、s__Alloiococcus_otitis、  s__Candidatus_Arthromitus_sp__SFB_mouse、  s__Candidatus_Arthromitus_sp__SFB_mouse_NL、s__Collinsella_ihuae、s__Dubosiella_newyorkensis、s__Enterococcus_faecalis、s__Faecalibaculum_rodentium、s__Fibrobacter_succinogenes、s__Kozakia_baliensis、s__Lactobacillus_hayakitensis、s__Lactobacillus_ruminis、s__Oceanobacillus_caeni、s__Olsenella_profusa、s__Olsenella_scatoligenes、s__Prevotella_paludivivens、s__Ruminococcus_faecis、s__Streptacidiphilus_sp__DSM_106435、s__Thiorhodococcus_drewsii | |
| Male experiment group | p__Fusobacteria、c__Fusobacteriia、o__Fusobacteriales、f__Fusobacteriaceae、f__Rhodobiaceae、g__Domibacillus、g__Ilyobacter、g__Mogibacterium、g__Solirubrum、s__Bacillus_cereus、s__Chryseobacterium_gallinarum、s__Domibacillus_enclensis、s__Ilyobacter_polytropus、s__Lactobacillus_plantarum、s__Microbacterium_hydrocarbonoxydans、s__Mogibacterium_pumilum、s__Solirubrum_puertoriconensis、s__Staphylococcus_nepalensis | |

Note: P denotes phylum, c denotes class, o denotes order, f denotes family, g denotes genus, and s denotes species.
