# Supplementary material for: Effect of Helicobacter pylori-related chronic gastritis on gastrointestinal microorganisms and brain neurotransmitters in mice
Source: Front Pharmacol. 2024 Dec 6;15:1472437. doi: 10.3389/fphar.2024.1472437 (PMC11659015; doi:10.3389/fphar.2024.1472437)
Supplement: Supplementary file 3 [file Table3.DOCX]

**Supplementary Table 3**

Gastrointestinal characteristic microorganisms of male and female mice in the control group.

| **Group** | **Gastrointestinal characteristic microorganisms（LDA > 2, *P* < 0.05）** |
| --- | --- |
| Female control group | p__Planctomycetes、c__Bacilli、c__Planctomycetia、o__Lactobacillales、  o__Mycoplasmatales、o__Planctomycetales、o__Xanthomonadales、  f__Bacillaceae、f__Lactobacillaceae、f__Mycoplasmataceae、  f__Peptoniphilaceae、f__Planctomycetaceae、f__Xanthomonadaceae、  g__Alkalitalea、g__Anoxybacillus、g__Apibacter、g__Bacillus、  g__Beduini、g__Celeribacter、g__Domibacillus、g__Fictibacillus、  g__Gemella、g__Granulicatella、g__Lactobacillus、g__Luteimonas、  g__Mariniblastus、g__Mycobacterium、g__Mycoplasma、g__Oceanobacillus、g__Peptoclostridium、g__Peptoniphilus、g__Provencibacterium、g__Pseudomonas、g__Turicibacter、g__Virgibacillus、s__Alkalitalea_saponilacus、s__Anoxybacillus_flavithermus、s__Apibacter_mensalis、s__Bacillus_abyssalis、s__Bacillus_alkalitelluris、s__Bacillus_cereus、s__Bacillus_eiseniae、s__Bacillus_ginsengihumi、s__Bacillus_humi、  s__Bacillus_massiliosenegalensis、s__Bacillus_subtilis、  s__Bacillus_wakoensis、s__Beduini_massiliensis、  s__Celeribacter_indicus、s__Clostridium_celatum、  s__Clostridium_disporicum、s__Fictibacillus_macauensis、  s__Gemella_asaccharolytica、s__Gemella_sanguinis、  s__Granulicatella_elegans、s__Lactobacillus_acetotolerans、  s__Lactobacillus_acidophilus、s__Lactobacillus_amylolyticus、  s__Lactobacillus_amylovorus、s__Lactobacillus_bifermentans、  s__Lactobacillus_crispatus、s__Lactobacillus_delbrueckii、  s__Lactobacillus_gallinarum、s__Lactobacillus_gasseri、  s__Lactobacillus_gigeriorum、s__Lactobacillus_hamsteri、  s__Lactobacillus_hominis、s__Lactobacillus_iners、  s__Lactobacillus_ingluviei、s__Lactobacillus_intestinalis、  s__Lactobacillus_jensenii、s__Lactobacillus_johnsonii、  s__Lactobacillus_kalixensis、s__Lactobacillus_parafarraginis、  s__Lactobacillus_paragasseri、s__Lactobacillus_plantarum、  s__Lactobacillus_rennini、s__Lactobacillus_reuteri、  s__Lactobacillus_salivarius、s__Listeria_ivanovii、  s__Listeria_weihenstephanensis、s__Luteimonas_abyssi、  s__Mariniblastus_fucicola、s__Muricauda_ruestringensis、  s__Mycoplasma_elephantis、s__Oceanobacillus_caeni、  s__Paenibacillus_ihumii、s__Paraburkholderia_sacchari、  s__Peptoclostridium_acidaminophilum、s__Peptoniphilus_sp__ING2_D1G、s__Provencibacterium_massiliense、s__Staphylococcus_hominis、s__Streptococcus_sanguinis、s__Turicibacter_sanguinis、s__Turicibacter_sp__H121、s__Virgibacillus_sp__Bac330 |
| Male control group | p__Verrucomicrobia、c__Gammaproteobacteria、c__Verrucomicrobiae、o__Enterobacterales、o__Verrucomicrobiales、f__Akkermansiaceae、  f__Alcaligenaceae、f__Christensenellaceae、f__Cytophagaceae、  f__Enterobacteriaceae、f__Microbacteriaceae、f__Morganellaceae、  f__Staphylococcaceae、g__Achromobacter、g__Akkermansia、  g__Atopobacter、g__Catenibacterium、g__Christensenella、g__Cytophaga、g__Escherichia、g__Gillisia、g__Globicatella、g__Levyella、g__Mariniphaga、g__Methylorubrum、g__Ohtaekwangia、g__Proteus、g__Staphylococcus、s__Achromobacter_xylosoxidans、  s__Akkermansia_muciniphila、s__Atopobacter_phocae、  s__Bacteroides_plebeius、s__Bifidobacterium_aesculapii、  s__Bifidobacterium_bifidum、s__Bosea_robiniae、  s__Catenibacterium_mitsuokai、s__Cytophaga_hutchinsonii、  s__Delftia_sp__HK171、s__Enterococcus_faecalis、s__Escherichia_coli、  s__Gillisia_sp__Hel1_33_143、s__Globicatella_sulfidifaciens、  s__Hymenobacter_coccineus、s__Lactobacillus_equi、  s__Levyella_massiliensis、s__Mariniphaga_anaerophila、  s__Methylorubrum_populi、s__Microbacterium_sp__PM5、  s__Oceanobacillus_sojae、s__Ohtaekwangia_koreensis、  s__Pontibacter_chinhatensis、s__Prevotella_ihumii、  s__Staphylococcus_aureus、s__Staphylococcus_sciuri、  s__Treponema_phagedenis |

Note: P denotes phylum, c denotes class, o denotes order, f denotes family, g denotes genus, and s denotes species.
