# Supplementary material for: Effect of Helicobacter pylori-related chronic gastritis on gastrointestinal microorganisms and brain neurotransmitters in mice
Source: Front Pharmacol. 2024 Dec 6;15:1472437. doi: 10.3389/fphar.2024.1472437 (PMC11659015; doi:10.3389/fphar.2024.1472437)
Supplement: Supplementary file 4 [file Table4.DOCX]

**Supplementary Table 4**

Gastrointestinal characteristic microorganisms of male and female mice in the experiment group.

| **Group** | **Gastrointestinal characteristic microorganisms（LDA > 2, *P* < 0.05）** |
| --- | --- |
| Female experiment group | p__Acidobacteria、p__Firmicutes、p__Ignavibacteriae、c__Acidithiobacillia、  c__Acidobacteria_subdivision_6、c__Deltaproteobacteria、c__Erysipelotrichia、c__Ignavibacteria、o__Acidithiobacillales、o__Desulfovibrionales、o__Erysipelotrichales、o__Halanaerobiales、o__Ignavibacteriales、f__Acidithiobacillaceae、f__Bacillaceae、f__Desulfomicrobiaceae、f__Desulfovibrionaceae、f__Erysipelotrichaceae、f__Halobacteroidaceae、f__Ignavibacteriaceae、f__Nannocystaceae、f__Vicinamibacteraceae、g__Acidithiobacillus、g__Allobaculum、g__Alloiococcus、g__Apibacter、g__Bacillus、g__Bhargavaea、g__Bilophila、g__Desulfitobacterium、g__Desulfomicrobium、g__Desulfovibrio、g__Desulfurispora、g__Dielma、g__Dubosiella、g__Faecalibaculum、g__Faecalicoccus、g__Fictibacillus、g__Gemella、g__Ignavibacterium、g__Luteitalea、g__Lysinibacillus、g__Oceanobacillus、g__Plesiocystis、g__Proteiniclasticum、g__Salinibacillus、g__Turicibacter、s__Acidithiobacillus_thiooxidans、s__Allobaculum_stercoricanis、s__Alloiococcus_otitis、s__Apibacter_mensalis、s__Atopobium_vaginae、s__Bacillus_abyssalis、s__Bacillus_alkalitelluris、s__Bacillus_drentensis、s__Bacillus_eiseniae、s__Bacillus_ginsengihumi、s__Bacillus_halosaccharovorans、s__Bacillus_humi、s__Bacillus_sonorensis、s__Bhargavaea_cecembensis、s__Bilophila_wadsworthia、s__Candidatus_Desulfovibrio_trichonymphae、s__Clostridium_acetireducens、s__Clostridium_celatum、s__Clostridium_chauvoei、s__Clostridium_gasigenes、s__Clostridium_homopropionicum、s__Clostridium_tepidiprofundi、s__Clostridium_tetani、s__Clostridium_ventriculi、s__Collinsella_tanakaei、s__Desulfitobacterium_hafniense、s__Desulfomicrobium_baculatum、s__Desulfovibrio_africanus、s__Desulfovibrio_cuneatus、s__Desulfovibrio_fairfieldensis、s__Desulfovibrio_gracilis、s__Desulfovibrio_piger、s__Desulfurispora_thermophila、s__Dielma_fastidiosa、s__Dubosiella_newyorkensis、s___Eubacterium__brachy、s__Faecalibaculum_rodentium、s__Faecalicoccus_pleomorphus、s__Fictibacillus_macauensis、s__Fusarium_venenatum、s__Gemella_sanguinis、s__Ignavibacterium_album、s__Lactobacillus_acetotolerans、s__Lactobacillus_amylolyticus、s__Lactobacillus_amylovorus、s__Lactobacillus_apis、s__Lactobacillus_bombicola、s__Lactobacillus_coleohominis、s__Lactobacillus_crispatus、s__Lactobacillus_equicursoris、s__Lactobacillus_floricola、s__Lactobacillus_gasseri、s__Lactobacillus_hamsteri、s__Lactobacillus_helveticus、s__Lactobacillus_hominis、s__Lactobacillus_intestinalis、s__Lactobacillus_kullabergensis、s__Lactobacillus_pasteurii、s__Lactobacillus_psittaci、s__Listeria_ivanovii、s__Listeria_weihenstephanensis、s__Luteitalea_pratensis、s__Mycolicibacterium_mageritense、s__Oceanobacillus_caeni、s__Oceanobacillus_senegalensis、s__Peptoniphilus_sp__ING2_D1G、s__Plesiocystis_pacifica、s__Proteiniclasticum_ruminis、s__Pseudomonas_aeruginosa、s__Salinibacillus_kushneri、s__Staphylococcus_hominis、s__Streptococcus_parauberis、s__Streptococcus_sanguinis、s__Turicibacter_sanguinis、s__Turicibacter_sp__H121、s__Virgibacillus_sp__Bac330、s__Xanthomonas_citri |
| Male experiment group | p__Chlorobi、c__Chlorobia、c__Gammaproteobacteria、o__Chlorobiales、  o__Enterobacterales、o__Neisseriales、o__Veillonellales、  f__Amoebophilaceae、f__Azonexaceae、f__Bacteroidaceae、f__Chlorobiaceae、  f__Cyclobacteriaceae、f__Enterobacteriaceae、f__Gottschalkiaceae、  f__Morganellaceae、f__Neisseriaceae、f__Symbiobacteriaceae、  f__Veillonellaceae、g__Algoriphagus、g__Bacteroides、  g__Candidatus_Arthromitus、g__Candidatus_Cardinium、g__Dechloromonas、  g__Escherichia、g__Mariniphaga、g__Megasphaera、g__Microbacterium、  g__Murdochiella、g__Neisseria、g__Nitratifractor、g__Prosthecochloris、  g__Proteus、g__Roseovarius、g__Simplicispira、g__Sphaerochaeta、  g__Symbiobacterium、s__Algoriphagus_antarcticus、s__Alistipes_inops、  s__Anaerotignum_propionicum、s__Bacteroides_caccae、  s__Bacteroides_caecimuris、s__Bacteroides_luti、s__Bacteroides_ovatus、  s__Bacteroides_paurosaccharolyticus、s__Bacteroides_stercoris、  s__Bifidobacterium_adolescentis、s__Bifidobacterium_asteroides、  s__Bifidobacterium_longum、s__Bifidobacterium_scardovii、  s__Bifidobacterium_thermophilum、s__Candidatus_Cardinium_hertigii、  s__Dechloromonas_sp__HYN0024、s__Domibacillus_enclensis、  s__Enterococcus_saccharolyticus、s__Escherichia_coli、s__Gillisia_limnaea、s__Hymenobacter_daecheongensis、s__Hymenobacter_sp__DG25A、s__Mariniphaga_anaerophila、s__Microbacterium_hydrocarbonoxydans、s__Neisseria_sp__KEM232、s__Nitratifractor_salsuginis、s__Prevotella_corporis、  s__Prevotella_oulorum、s__Prosthecochloris_sp__GSB1、s__Proteus_vulgaris、  s__Simplicispira_psychrophila、s__Sphaerochaeta_globosa、  s__Staphylococcus_nepalensis、s__Symbiobacterium_thermophilum |

Note: P denotes phylum, c denotes class, o denotes order, f denotes family, g denotes genus, and s denotes species.
